# Supplementary material for: Reconstruction of a generic genome-scale metabolic network for chicken: Investigating network connectivity and finding potential biomarkers
Source: PLoS One. 2022 Mar 22;17(3):e0254270. doi: 10.1371/journal.pone.0254270 (PMC8939822; doi:10.1371/journal.pone.0254270)
Supplement: Attachment — Submitted filename: comments.docx [file pone.0254270.s003.docx]

**In abstract**- Line 16 - mention which database has been used.

**In Introduction**- Please use newly published paper up to 5 years in the introduction, it is highly informative for Reconstruction of a generic genome-scale metabolic network for chicken.

Line 34 - mention which models have a black box nature

The authors do not manage well the importance of *Gallus gallus* metabolism

**In Methodology section-** 2.1 Add more details about the databases mentioned

Line 92- KEGG, BiGG and CHEBI explain them

Line 95- The intracellular pH of 7.2 was considered on which basis?

Section- 2.2 Add more details about COBRA toolbox

Line 120-121 for which purpose RPMI-like culture medium was used?

Section 2.3 line 135 you mentioned “According to the method presented by” kindly elaborate the method on which basis sick lethal and single gene deletion specified.

Section 2.4 line 147 you used term “Array Express” is it a database or software? Elaborate

Line 161: Motivate/Clarify why a threshold of 0.25 and cutoff of 0.9 was used?

Section 2.5 the algorithm of Principal Component Analysis is based on a few mathematical ideas. Explain which mathematical ideas have been used for both algorithms GIMME and TRFBA?

**In results section-** 3.1 mention more details about gene associated reactions and non-gene associated reactions.

Line 183- Define the gap-filling process.

Line 233-234 The PCA results also indicated that the normal chicken cell was the most different cell line from the other cell lines mentions other cell lines also by name

Overall improve the quality of figures especially redraw the figure 2.

**In conclusion section-** add future perspectives that came from your very interesting and well wrote paper
