# Supplementary material for: Reconstruction of a generic genome-scale metabolic network for chicken: Investigating network connectivity and finding potential biomarkers
Source: PLoS One. 2022 Mar 22;17(3):e0254270. doi: 10.1371/journal.pone.0254270 (PMC8939822; doi:10.1371/journal.pone.0254270)
Supplement: Attachment — Submitted filename: Response to Reviewers.docx [file pone.0254270.s004.docx]

**Journal of PLOS ONE**

Manuscript ID PONE-D-21-17962

Title: *Reconstruction of a generic genome-scale metabolic network for chicken: investigating network connectivity and finding potential biomarkers*

**Response to reviewer’s comment and questions**

We would like to greatly appreciate your attention to our manuscript. We acknowledge that our manuscript is reinforced after the revisions applied to the manuscript based on the constructive comments received from the professional editor and reviewer. As requested, the questions and comments are answered and addressed below meticulously. We also used both highlighting and track changes. The edited manuscript is attached, as well. We hope the major and minor revisions serve to draw your attention to find the manuscript worthy of publication.

Thank you again for perusing this context. We would welcome any further specific questions you might have.

**Comments from the Academic Editor**

1. Please ensure that your manuscript meets PLOS ONE's style requirements, including those for file naming.

Regarding the PLOS ONE’s format requirements, we double-checked all parts of the manuscript.

2. We suggest you thoroughly copyedit your manuscript for language usage, spelling, and grammar. If you do not know anyone who can help you do this, you may wish to consider employing a professional scientific editing service.

We assure the editor that our manuscript has been edited for language by the native English editorial board of Research Editor. The certification will be submitted along with the other files.

3. Please upload a new copy of Figures 1, 2, 3 and 4 as the detail is not clear. Please follow the link for more information.

We changed some details of each figure for a better review. We also used Preflight Analysis and Conversion Engine (PACE) digital diagnostic tool as you recommended to ensure that figures meet PLOS requirements.

4. Please review your reference list to ensure that it is complete and correct. If you have cited papers that have been retracted, please include the rationale for doing so in the manuscript text, or remove these references and replace them with relevant current references. Any changes to the reference list should be mentioned in the rebuttal letter that accompanies your revised manuscript. If you need to cite a retracted article, indicate the article’s retracted status in the References list and also include a citation and full reference for the retraction notice.

All references were double-checked. There was not any retracted article to mention or remove. The following references were added to the manuscript regarding the reviewers’ comments to make the content of the article more valuable:

- Ref #4: Stop explaining black box machine learning models for high stakes decisions and use interpretable models instead.
- Ref #24: Early Growth and Protein-Energy Metabolism in Chicken Lines Divergently Selected on Ultimate pH.
- Ref #25: Chicken Is a Useful Model to Investigate the Role of Adipokines in Metabolic and Reproductive Diseases.
- Ref #26: Nutrition and metabolism in poultry: role of lipids in early diet.
- Ref #33: MetaNetX / MNXref : unified namespace for metabolites and biochemical reactions in the context of metabolic models.
- Ref #43: Chicken Immune Cell Assay to Model Adaptive Immune Responses In Vitro.
- Ref #44: Cryopreservation, and Mitogenesis of Peripheral Blood Lymphocytes from Chickens (Gallus domesticus) and Wild Herring Gulls (Larus argentatus)
- Ref #45: In vitro Chicken Bone Marrow-Derived Dendritic Cells Comprise Subsets at Different States of Maturation.
- Ref #56: ArrayExpress update – from bulk to single-cell expression data.
- Ref #60: Systematic Evaluation of Methods for Integration of Transcriptomic Data into Constraint-Based Models of Metabolism.
- Ref #62: Systematizing the generation of missing metabolic knowledge.

The following references were also removed from the former version of manuscript in order to replace with newer references:

- Ref #23: Metabolic disorders: Limitations to growth of and mineral deposition into the broiler skeleton after hatch and potential implications for leg problems.
- Ref #49: ArrayExpress update-simplifying data submissions.

**Comments from the 1^st^ Reviewer:**

Your interest of research is good. Revise your manuscript accordingly and revise the grammatical errors in the paper explain all the possible terms mentioned in the paper as they are described not properly through the manuscript.

According to reviewer’s comment, all parts of the paper were double-checked grammatically.

1. In“2.1, The procedure of genome-scale model reconstruction for *gallus gallus*” part, it will be helpful to explain the rules and the basis for adding new name for those reactions or metabolite which have no name.

Regarding the naming of reactions and metabolites, we did our best to make the model naming most similar to the BiGG database to conform to community standards [1]. In some cases that we did not find the reactions of metabolic pathways in BiGG, KEGG reaction IDs were used [2]. For transport reactions, we used the MetaNetX database identifiers [3]. More explanations were added to the section.

2. In “2.2 In silico simulations condition”, is there any reference for the setting of the reaction upper and lower bounds?

Conventionally, all reactions in metabolic models must have infinite lower and upper bounds to accept any flux. This infinity is shown by setting 1000$\frac{mmol}{gDCW.h}$. For exchange reactions, these bounds have to be adjusted based on the culture media conditions. As mentioned in the manuscript, we used RPMI-like media to change the lower and upper bounds of the exchange reactions [4]; and for those that were not participated in RPMI-like media, their lower bounds were set to zero. More explanations were added to the manuscript related to this section.

3. In “3.1 Characteristics of the reconstructed model” part, it is better to add the calculation of network correlation to illustrate the similarity between the networks.

In figure 2d, wherein four metabolic models were compared, no specific calculations were used. To draw this part of the figure, we counted the number of reactions that had gene to reaction association and divided it by the number of all reactions in each model. However, we acknowledge that the explanations in this section may need to be added in order to avoid confusion. As a result, we added a description to this section.

4. In the discussion part, it's suggested to include some critical thinking and exemplify the application of this research.

The reconstructed model can be used as a platform for other researchers to broaden their knowledge and studies of chicken cells' metabolic interactions more efficiently. In addition to a general platform, we also highlighted potential biomarkers that drugs can target to avoid chickens' common diseases. More explanations have been added to the manuscript to give this study a better and deeper mindset.

5. Please pay attention to the inconsistency in font and case, such as “by” in line 234, and “Metabolisms” in line 267.

We thank the reviewer for notifying us about some inconsistencies in the manuscript. We	assure we have re-checked the whole manuscript so that there are no more	inconsistencies in fonts or cases again.
**Comments from the 2^nd^ reviewer:**

In this manuscript, the authors reconstructed a generic genome-scale metabolic network for chicken named iES1300, which was consists of 2427 reactions, 2526 metabolites, and 1300 genes. In addition, they compared the iES1300 with three other important models to evaluate the interaction of metabolic gene networks. Meanwhile, they found several important biomarkers based on PCA. Overall, this work is meaningful and well-organized.

However, the whole manuscript has bioinformatics results only. In order to illustrate the reliability of those biomarkers, in vitro or in vivo biochemical verification is suggested.

Despite our efforts to validate some parts of our study experimentally, there was a lack of lab materials such as related cell lines for chicken and also culture media to grow it in Iran. Instead, we utilized other well-known experiment results in which microarray and RNA sequencing technologies have been performed to generate raw transcriptomic data. We then computationally processed these experimental raw data and analyzed them by integrating them with our model.

**In abstract**- Line 16 - mention which database has been used.

All databases used in our study were added to the abstract section.

**In Introduction**- Please use newly published paper up to 5 years in the introduction, it is highly informative for Reconstruction of a generic genome-scale metabolic network for chicken.

The authors appreciate the reviewer’s comment; many of our references in this section are from 2014 onwards; however, a few articles were older but extremely important to be cited in the paper. We assure reviewers that we added newer papers to the introduction, especially regarding the importance of chicken metabolism.

Line 34 - mention which models have a black box nature:

Behavior or understanding the models derived by machine learning algorithms is sometimes tough to comprehend or interpret, especially deep neural networks. These ML systems are considered examples of black-box models [5]. There may be a question for those who read our paper why we chose a constraint-based reconstruction approach instead of machine learning algorithms. Therefore, we tried to clarify our preference for choosing a constraint-based reconstruction over a machine learning approach.

The authors do not manage well the importance of *Gallus gallus* metabolism

As a potential source of protein, many efforts have been made for both embryonic and adult chickens, ranging from the growth rate to meat yield, and the feeding strategies and efficiencies [6–8]. Studies have shown that breast muscle's *in vivo* glycogen content correlates with meat quality [6]. On the other hand, it has been reviewed that continuous progress in optimizing meat and egg production has led to various disorders in metabolism and reproduction; then it has negative implications on humans as a result of consuming chicken's meat and egg [7]. These obstacles can be overcome by diving more into chicken metabolism. The whole body paragraph related to the importance of the chicken metabolism were rearranged, and these explanations were added.

**In Methodology section-** 2.1 Add more details about the databases mentioned

Line 92- KEGG, BiGG and CHEBI explain them

To provide the draft of the model, the KEGG database was used to extract biological information of each *gallus gallus* metabolic pathway, mainly including enzymes and reactions [2]. These reaction and metabolite IDs were used from BiGG database to conform to community standards [1]. We also check metabolites and their formula and charge with further investigation on the CHEBI database [9]. These explanations were added.

Line 95- The intracellular pH of 7.2 was considered on which basis?

Our claim pf setting pH values of 7.2 can be proved by several studies related to chicken’s intracellular pH [10]. This is a typical pH value in the reconstruction of metabolic networks [11].

Section- 2.2 Add more details about COBRA toolbox:

COBRA toolbox is a MATLAB package and has been developed to implement constraint based reconstruction and analysis methods; i.e. it allows researchers to infer and analyze reconstructed models [12]. In this way, COBRA toolbox has the capability of converting biological data into a mathematical model, then evaluate models by defining or changing objective functions, and improve the reconstructed model by deploying gap-filling process [12].

Line 120-121 for which purpose RPMI-like culture medium was used?

To simulate growth conditions, we needed a well-known culture medium by which we can evaluate the model's performance whether it grows or not. To this end, we limited exchange reactions correlated to the RPMI-like medium. More explanations have been added to the manuscript in “*In silico* simulation condition” section.

Section 2.3 line 135 you mentioned “According to the method presented by” kindly elaborate the method on which basis sick lethal and single gene deletion specified.

Single Gene Deletion is one of the COBRA toolbox analysis modules and calculates the effect of each gene on the cell growth one-by-one [12]. Next, according to Motamedian et al. method [13], we defined a threshold on these growth rate ratios. In such a way that this ratio was lower than 10^-5^ for a particular gene, it must be a lethal gene, and if this ratio was just below 1 for other specific genes, it meant that those genes are categorized as sick genes.

Section 2.4 line 147 you used term “Array Express” is it a database or software? Elaborate

ArrayExpress is one of the largest online databases that acts as an archive of functional genomics data [14]. It was founded for microarray datasets at first, but the number of datasets submitted based on sequencing experiments has surprisingly been exceeded [14].

Line 161: Motivate/Clarify why a threshold of 0.25 and cutoff of 0.9 was used?

The threshold value was set to the 25th percentile of the given expression data to inactivate reactions below a specific mRNA transcript level based on Machado and Herrgard study [15]. The second parameter for the objective function flux cutoff was used according to the original paper, i.e., the GIMME output context-specific metabolic network was forced to grow no less than 90% of the maximum growth [16]. We applied these explanations to the manuscript to avoid confusion.

Section 2.5 the algorithm of Principal Component Analysis is based on a few mathematical ideas. Explain which mathematical ideas have been used for both algorithms GIMME and TRFBA?

GIMME and TRFBA are both integration algorithms and can integrate omics data with a general metabolic model to create a context-specific one. GIMME is a discrete algorithm to find and remove the reactions that correlate with lower gene expression levels by adjusting the two parameters of cutoff and threshold, which were mentioned earlier [16]. On the other hand, TRFBA is a continuous algorithm tries to regulate the upper bounds of the model reactions by assigning a constant parameter, which is known as C [17]. Therefore, we hypothesized that compiling both algorithms differentiate cell lines with a higher confidence level [18].

**In results section-** 3.1 mention more details about gene associated reactions and non-gene associated reactions.

None-gene associated reactions, also known as orphan reactions, are those that have not been identified which genes or proteins are encoding them [19], but we added them to the model so that the model can grow. On the contrary, gene associated reactions are reactions for which a gene or several genes have been assigned [19].

Line 183- Define the gap-filling process.

Even the most comprehensive reconstructed models with highest confidence scores may have their own deficiencies [19]. This is mainly due to inadequate knowledge of the metabolism which leads to missing metabolites and reactions. For this reason, gap-filling is needed to refine the model inconsistencies [19]. To this end, for the growth of the chicken cell, we checked each component in the biomass production reaction to make sure they are not dead-end metabolites and see if they carry flux.

Line 233-234 The PCA results also indicated that the normal chicken cell was the most different cell line from the other cell lines mentions other cell lines also by name

Other cell lines were added also by name, as the reviewer suggested.

Overall improve the quality of figures especially redraw the figure 2.

The quality of all figures were improved, as the reviewer and the editor recommended.

**In conclusion section-** add future perspectives that came from your very interesting and well wrote paper

The authors appreciate the reviewer’s comments. We added more details about future perspectives of our study in the conclusion section by exemplifying the applications of this research.

References

1.	King ZA, Lu J, Dräger A, Miller P, Federowicz S, Lerman JA, et al. BiGG Models: A platform for integrating, standardizing and sharing genome-scale models. Nucleic Acids Res. 2016;44: D515–D522. doi:10.1093/nar/gkv1049

2.	Kanehisa M, Sato Y, Furumichi M, Morishima K, Tanabe M. New approach for understanding genome variations in KEGG. Nucleic Acids Res. 2019;47: D590–D595. doi:10.1093/nar/gky962

3.	Tran VDT, Mehl F, Ibberson M, Pagni M. MetaNetX / MNXref : unified namespace for metabolites and biochemical reactions in the context of metabolic S ebastien. 2021;49: 570–574. doi:10.1093/nar/gkaa992

4.	Folger O, Jerby L, Frezza C, Gottlieb E, Ruppin E, Shlomi T. Predicting selective drug targets in cancer through metabolic networks. Mol Syst Biol. 2011;7: 501. doi:10.1038/msb.2011.35

5.	Rudin C. Stop explaining black box machine learning models for high stakes decisions and use interpretable models instead Cynthia. Nat Mach Intell. 2019;1: 206–215. doi:10.1038/s42256-019-0048-x

6.	Métayer-Coustard S, Tesseraud S, Praud C, Royer D, Bordeau T, Coudert E, et al. Early Growth and Protein-Energy Metabolism in Chicken Lines Divergently Selected on Ultimate pH. Front Physiol. 2021;12. doi:10.3389/fphys.2021.643580

7.	Mellouk N, Ramé C, Barbe A, Grandhaye J, Froment P, Dupont J. Chicken Is a Useful Model to Investigate the Role of Adipokines in Metabolic and Reproductive Diseases. Luconi M, editor. Int J Endocrinol. 2018;2018: 4579734. doi:10.1155/2018/4579734

8.	Cherian G. Nutrition and metabolism in poultry: role of lipids in early diet. J Anim Sci Biotechnol. 2015;6: 28. doi:10.1186/s40104-015-0029-9

9.	Hastings J, De Matos P, Dekker A, Ennis M, Harsha B, Kale N, et al. The ChEBI reference database and ontology for biologically relevant chemistry: Enhancements for 2013. Nucleic Acids Res. 2013;41. doi:10.1093/nar/gks1146

10.	Oginuma M, Harima Y, Tarazona OA, Diaz-cuadros M. Intracellular pH controls WNT downstream of glycolysis in amniote embryos. 2020. doi:10.1038/s41586-020-2428-0

11.	Thiele I, Palsson B. A protocol for generating a high-quality genome-scale metabolic reconstruction. Nat Protoc. 2010;5: 93–121. doi:10.1038/nprot.2009.203

12.	Schellenberger J, Que R, Fleming RMT, Thiele I, Orth JD, Feist AM, et al. Quantitative prediction of cellular metabolism with constraint-based models: The COBRA Toolbox v2.0. Nat Protoc. 2011;6: 1290–1307. doi:10.1038/nprot.2011.308

13.	Motamedian E, Ghavami G, Sardari S. Investigation on metabolism of cisplatin resistant ovarian cancer using a genome scale metabolic model and microarray data. Iran J Basic Med Sci. 2015;18: 267–276. doi:10.22038/ijbms.2015.4131

14.	Athar A, Anja F, George N, Iqbal H, Huerta L, Ali A, et al. ArrayExpress update – from bulk to single-cell expression data. 2019;47: 711–715. doi:10.1093/nar/gky964

15.	Machado D, Herrgård M. Systematic Evaluation of Methods for Integration of Transcriptomic Data into Constraint-Based Models of Metabolism. PLOS Comput Biol. 2014;10: 1–12. doi:10.1371/journal.pcbi.1003580

16.	Becker SA, Palsson BO. Context-specific metabolic networks are consistent with experiments. PLoS Comput Biol. 2008;4. doi:10.1371/journal.pcbi.1000082

17.	Motamedian E, Mohammadi M, Shojaosadati SA, Heydari M. TRFBA: An algorithm to integrate genome-scale metabolic and transcriptional regulatory networks with incorporation of expression data. Bioinformatics. 2017;33: 1057–1063. doi:10.1093/bioinformatics/btw772

18.	Jamialahmadi O, Hashemi-Najafabadi S, Motamedian E, Romeo S, Bagheri F. A benchmark-driven approach to reconstruct metabolic networks for studying cancer metabolism. PLoS Comput Biol. 2019;15. doi:10.1371/journal.pcbi.1006936

19.	Orth JD, Palsson BØ. Systematizing the generation of missing metabolic knowledge. Biotechnol Bioeng. 2011;107: 403–412. doi:10.1002/bit.22844.Systematizing
